# The accuracy of different calculation methods when identifying handgrip strength asymmetry among middle-aged and older Chinese adults

**DOI:** 10.1371/journal.pone.0299469

**Published:** 2024-03-28

**Authors:** Yilin Wang, Jing Wang, Binyou Wang, Jing Fu, Xiaoyan Chen

**Affiliations:** 1 Department of Geriatric Medicine, Zigong Mental Health Center, the Zigong Affiliated Hospital of Southwest Medical University, Zigong, Sichuan Province, China; 2 School of Nursing, Southwest Medical University, Luzhou, Sichuan Province, China; 3 Deyang People’s Hospital, Deyang, Sichuan Province, China; Mexican Social Security Institute: Instituto Mexicano del Seguro Social, MEXICO

## Abstract

At present, there is no uniform standard mean of identifying handgrip strength (HGS) asymmetry based on maximum or average HGS values. Therefore, this study aimed to explore the accuracy of different calculation methods in the evaluation of HGS asymmetry. Using the maximum reading of two trials from both hands (Method A) as the reference standard, the accuracy of the HGS asymmetry identified by the average value of two trials of both hands (Method B) was determined by using various indicators, including specificity, sensitivity, the area under the receiver operating characteristic curve (AUC), positive, and negative predictive values. Overall, 12,163 individuals were included in this study, of whom 47.61% (5791/12,163) were male. The percentages of individuals with HGS asymmetry differed as a function of age and sex when using these two different methods. When employing Method A, 38.52%, 41.57%, and 44.57% of males 45 ≤ age<60, 60 ≤ age<80, and ≥ 80 years of age exhibited HGS asymmetry as compared to 40.78%, 39%, and 39.63% of females. Using Method B, the corresponding proportions were 41.69%, 42.5%, and 40% in males and 42.01%, 41.18%, and 40.55% in females, respectively. When compared to Method A, Method B was found to be effective in identifying HGS asymmetry, with AUC values ranging from 0.844 to 0.877. However, there was only moderate agreement between the two methods in assessing HGS asymmetry. Specifically, the Kappa values for the two Methods were 0.692, 0.694, and 0.766 in males aged 45 to 60, 60 to 80, and 80 years and above, respectively. For females, the Kappa values were 0.674, 0.661, and 0.751, respectively. These results demonstrated that the maximal or average HGS values from two trials using both hands has a significant impact on the consequent identification of HGS asymmetry.

## Introduction

Handgrip strength (HGS) asymmetry is a novel HGS indicator defined by a ratio of non-dominant to dominant HGS that is greater than 1.1 or less than 0.9 [1] and it may reflect differences in neuromuscular system function or brain health [2]. HGS asymmetry is reported to be highly prevalent among middle-aged and older adults [3–6]. Recent studies have demonstrated that individuals with asymmetric HGS experience worse health outcomes, such as chronic morbidity [7], limitations in individual basic self-care tasks [8], increased risk of multimorbidity [9], higher odds of developing neurodegenerative disorders [5], greater incidence of falls [3], more rapid mortality [10], and lower cognitive function [11]. As such, it is vital that HGS asymmetry be identified as early as possible to provide appropriate interventions.

Currently, there are two procedures that are commonly used for the evaluation of HGS. The 2019 guidelines of the Asian Working Group for Sarcopenia (AWGS) suggest that HGS should be measured using the highest recorded values obtained from either both hands or the dominant hand [12]. Conversely, the revised guidelines of the American Society of Hand Therapists (ASHT) recommends calculating the average measurement for each hand [13]. Thus, to date, there is no uniform standard regarding the identification of HGS. Consequently, there is no consistent definition for HGS asymmetry, as it is typically described as a condition where the HGS of one hand is more than 10% stronger than that of the other.

Both of these HGS calculation methods have been used across different studies [9, 11, 14, 15]. For example, to explore the association between HGS asymmetry and cognitive performance in older adults, a recent study enrolled 2729 Americans over 60 years of age and adopted the average HGS method [14], while an earlier study enrolled 17,163 Americans over 65 years of age and used the maximal HGS method [11]. Notably, the effect of this choice between the use of maximal or average HGS values on HGS asymmetry remains unknown.

This study was thus developed to assess whether the choice of maximum or average HGS value affects the detection of HGS asymmetry, and the relative accuracy of approaches to detecting HGS asymmetry. As HGS is influenced by age [16] and males generally have significantly higher HGS than females [17], we thus stratified the agreement analyses for the two HGS calculation methods based on participant age and sex.

## Methods

### Ethics statement

The data used to conduct this study were derived from the China Health and Retirement Longitudinal Study (CHARLS), an ongoing longitudinal survey for adults over the age of 45 in China. Data collection received ethical approval from the Peking University (IRB00001052-11015), which was the original CHARLS research team. As the data are public, the Ethics Committee of Zigong Mental Health Center (IRB number: 2023041004) waived the requirement for informed consent by the participants.

### Study design and participant characteristics

This observational study did not involve patient contact, was anonymous, and did not include any clinical intervention. For the present study, data were used from participants that: (1) were 45+ years of age at Wave one (2011), (2) self-reported hand dominance, and (3) underwent two rounds of HGS testing for both their dominant and non-dominant hands. Participants were excluded from the study if they did not report their hand dominance, if they only had one measurement available for either the dominant or non-dominant hand, or if they had obviously incorrectly recorded data. Examples of obviously incorrect records such as height (cm) measurements of 1.56, 1.64, 1.72, or 993, and weight (kg) measurements of 4 or 0.

### HGS asymmetry

The HGS values were measured when the participants were standing (or sitting, if unable to stand unaided) with their elbows bent to 90°. The HGS was measured using a dynamometer (EH101; CAMRY, Guangdong, China), maintained under specified conditions according to the instructions by trained technicians. The dynamometer had a range of 0 to 90.0 kg. The HGS testing was begun with the non-dominant hand, followed by the dominant hand. A total of two tests were conducted, and participants were given ample time to rest between the tests until they felt ready to proceed with the next round of testing. The maximal reading calculated from two tests was used to reflect HGS (standard, Method A), and the HGS value was also calculated from the average value from these two tests (Method B). The HGS ratio was calculated as non-dominant HGS to dominant HGS. In cases where this HGS ratio was below 1, the HGS ratio quotient (1/HGS ratio) was computed to standardize all HGS ratios such that HGS ratios of 0.9 and 1.1 were equivalent to one another. HGS asymmetry was defined as an HGS ratio greater than 1.1.

### Covariates

Covariates information was obtained from the Wave one data from the CHARLS study, including age, height, weight, marital status (Married or Single/Divorced/Widowed), education (illiterate, primary school and below, junior high school and above), drinking history, smoking history, chronic lung diseases, hypertension, diabetes, heart diseases, dyslipidemia, kidney disease, dominant hand, stroke, arthritis or rheumatism, cancer or malignancies.

### Statistical analyses

In this study, body mass indices (BMIs) are reported as medians (P25, P75) due to the non-normal distribution of the data, while categorical variables are presented as numbers and percentages. Baseline characteristic comparisons were performed using rank-sum and Pearson’s chi-square tests. Differences in the identification of HGS asymmetry between HGS calculation methods were analyzed using Pearson chi-square tests. The discrepancies in HGS asymmetry and low HGS identified using different calculation methods were represented by Kappa values.

Using the maximal reading calculated from two tests (Method A) as the reference standard, the diagnostic accuracy of Method B when evaluating HGS asymmetry and low HGS was assessed based on the area under the receiver operating characteristic curve (AUC), sensitivity, specificity, and false positive and negative rates.

Data were analyzed using SPSS 25.0 (IBM Corp, Armonk, NY, USA). A two-sided P < 0.05 was the cut-off for statistical significance.

## Results

### Participant characteristics

We enrolled 12,163 participants (5791 males, 6372 females), of whom 92.81% were right-handed. Table 1 summarizes the characteristics of these participants. Women exhibited significantly more proportions of younger and older individuals than males in this study (3644 females vs. 3037 males in the 45–59 age group; 217 females vs. 175 males in the over-80 age group; P<0.001), were more prone to heart disease (P = 0.041), and had higher BMIs (P<0.001). Moreover, men were more prone to drinking than women in this study cohort (P<0.001).

**Table 1:** Baseline characteristics of the participants.

Characteristic	Male	Female	P
	(N = 5791)	(N = 6372)	
**Age,year,n(%)**			<0.001
45–59	3037(45.5)	3644(54.5)	
60–79	2579(50.7)	2511(49.3)	
≥80	175(44.6)	217(55.4)	
**Marital status, n(%)**			0.351
Married	5060(47.8)	5536(52.2)	
Divorced/Widow	691(47.0)	778(53.0)	
Single	40(40.8)	58(59.2)	
**Education,n(%)**			0.169
Illiterate	1651(48.8)	1731(51.2)	
Primary school and below	2346(47.3)	2614(52.7)	
Junior high school and above	1793(47.0)	2022(53.0)	
**Smoking,n(%)**			0.792
No	3511(47.5)	3877(52.5)	
Yes	2279(47.8)	2492(52.2)	
**Drinking,n(%)**			<0.001
No	4163(44.0)	5309(56.0)	
Yes	1628(60.5)	1063(39.5)	
**Chronic lung diseases,n(%)**			0.845
No	5164(47.7)	5670(52.3)	
Yes	605(47.4)	672(52.6)	
**Hypertension,n(%)**			0.947
No	4367(47.6)	4807(52.4)	
Yes	1393(47.7)	1529(52.3)	
**Diabetes,n(%)**			0.635
No	5423(47.7)	5952(52.3)	
Yes	315(46.7)	359(53.3)	
**Heart disease,n(%)**			0.041
No	5123(48.0)	5559(52.0)	
Yes	636(45.1)	775(54.9)	
**Dyslipidemia,n(%)**			0.285
No	5199(47.9)	5657(52.1)	
Yes	488(46.2)	569(53.8)	
**Kidney disease,n(%)**			0.73
No	5374(47.6)	5921(52.4)	
Yes	378(48.2)	406(51.8)	
**Stroke disease,n(%)**			0.77
No	5670(47.6)	6236(52.4)	
Yes	104(46.6)	119(53.4)	
**Cancer or malignant,n(%)**			0.287
No	5714(47.6)	6283(52.4)	
Yes	60(52.6)	54(47.4)	
**Arthritis or Rheumatism,n(%)**			0.575
No	3792(47.4)	4202(52.6)	
Yes	1987(48.0)	2155(52.0)	
**Dominant hand,n(%)**			0.445
Left	427(48.9)	447(51.1)	
Right	5364(47.5)	5925(52.5)	
**BMI, kg/m** ^**2**^ **,median(p25, p75)**	22.5(20.4,25.0)	23.6(21.2,26.3)	<0.001

### Differences in the identification of HGS asymmetry between different calculation methods

Table 2 highlights differences in the identification of HGS asymmetry between the two calculation methods as stratified by sex and age. Different proportions of individuals with HGS asymmetry were identified when using these two methods as a function of age groups and sex. When employing Method A, 38.52% (1170/3037), 41.57% (1072/2579), and 44.57% (78/175) of males 45 ≤ age<60, 60 ≤ age<80, and ≥ 80 years of age exhibited HGS asymmetry as compared to 40.78% (1486/3644), 39% (979/2511), and 39.63% (86/217) of females. Using Method B, the corresponding proportions were 41.69% (1266/3037), 42.5% (1096/2579), and 40% (70/175) in males and 42.01% (1531/3644), 41.18% (1034/2511), and 40.55% (88/217) in females, respectively (Table 2). The rates of HGS asymmetry identified using Method B were higher than those for Method A irrespective of participant age or sex (all P<0.001).

**Table 2:** Differences in the identification of asymmetric HGS according to the calculation method.

Variables				Method A		P-value
				Normal	Asymmetry	
**Method B**	**Male**	45≤age<60	Normal	1594(90.0)	177(10.0)	<0.001
			Asymmetry	273(21.6)	993(78.4)	
		60≤age<80	Normal	1303(87.9)	180(12.1)	<0.001
			Asymmetry	204(18.6)	892(81.4)	
		80≤age	Normal	91(86.7)	14(13.3)	<0.001
			Asymmetry	6(8.6)	64(91.4)	
	**Female**	45≤age<60	Normal	1847(87.4)	266(12.6)	<0.001
			Asymmetry	311(20.3)	1220(79.7)	
		60≤age<80	Normal	1300(88.0)	177(12.0)	<0.001
			Asymmetry	232(22.4)	802(77.6)	
		80≤age	Normal	117(90.7)	12(9.3)	<0.001
			Asymmetry	14(15.9)	74(84.1)	

Note: HGS = handgrip strength; Method A = max HGS value of both hands; Method B = average HGS value of both hands.

### Comparisons of the accuracy of different approaches to detecting HGS asymmetry

The diagnostic accuracy associated with the use of different HGS calculation methods as a means of detecting HGS asymmetry is detailed in Table 3. When using Method A as the reference standard, the use of Method B in the three defined age groups (45 ≤ age<60, 60 ≤ age<80, and ≥ 80 years) was associated with AUC values from 0.868 (95%CI: 0.851–0.885) to 0.877 (95%CI: 0.862–0.892), specificity values from 85.4% - 93.8%, sensitivity values from 82.1% - 84.9%, and Kappa values from 0.692–0.766 among males. Similarly, in females Method B exhibited AUC values from 0.846 (95%CI: 0.827–0.865) to 0.876 (95%CI: 0.816–0.936), specificity values from 81.9% - 89.3%, sensitivity values from 82.1% - 86%, and Kappa values from 0.661–0.751.

**Table 3:** Diagnostic accuracies of different HGS values for the determination of HGS asymmetry.

Variables	Cutoff	Specificity	Sensitivity	AUC	PPV	NPV	Kappa value
**Male** *							
Maximum value of both hands	1.1	Ref.	Ref.	Ref.	Ref.	Ref.	Ref.
Average value of both hands	1.1	85.4%	84.9%	0.877(0.862–0.892)	0.78	0.90	0.692
**Male** **							
Maximum value of both hands	1.1	Ref.	Ref.	Ref.	Ref.	Ref.	Ref.
Average value of both hands	1.1	86.5%	83.2%	0.868(0.851–0.885)	0.81	0.88	0.694
**Male** ***							
Maximum value of both hands	1.1	Ref.	Ref.	Ref.	Ref.	Ref.	Ref.
Average value of both hands	1.1	93.8%	82.1%	0.875(0.911–0.94)	0.91	0.87	0.766
**Female** *							
Maximum value of both hands	1.1	Ref.	Ref.	Ref.	Ref.	Ref.	Ref.
Average value of both hands	1.1	85.6%	82.1%	0.865(0.850–0.879)	0.8	0.87	0.674
**Female** **							
Maximum value of both hands	1.1	Ref.	Ref.	Ref.	Ref.	Ref.	Ref.
Average value of both hands	1.1	81.9%	84.9%	0.846(0.827–0.865)	0.78	0.88	0.661
**Female** ***							
Maximum value of both hands	1.1	Ref.	Ref.	Ref.	Ref.	Ref.	Ref.
Average value of both hands	1.1	89.3%	86.0%	0.876(0.816–0.936)	0.84	0.91	0.751

Note

*45≤age<60

**60≤age<80

***80≤age

HGS: handgrip strength

## Discussion

This study is the first to compare the identification of HGS asymmetry using both maximal and average values for both hands. Our findings indicated that the rate and consistency of identifying HGS asymmetry differed significantly according to the calculation method used. Regardless of sex and age, the rates of HGS asymmetry identified using average values for both hands were higher compared to those identified using maximal values for both hands. Furthermore, the identification of asymmetric HGS using the average method showed only moderate consistency with the method using the maximum.

Previous studies have reported a high rate of HGS asymmetry among older adults worldwide. It has been found to affect approximately 53.5% of Americans [3] and 45.6% of South Koreans [4]. However, the present study revealed a relatively lower rate of HGS asymmetry among Chinese middle-aged and older adults. Using the maximal HGS calculation method, it was found that 40% of participants exhibited HGS asymmetry, while using the average HGS calculation method, the rate was slightly higher at 41.8%.

Although the mechanistic basis for HGS asymmetry is still unclear, there are several potential causes including neurodegenerative disorders [5], motoric cognitive risk syndrome [18], brain hemisphere morbidity-related dysfunction [11], overcompensation, and mechanical deficits due to acute or chronic injuries [19]. Therefore, the lower frequency of adults with HGS asymmetry in this population may be attributable to the fact that (1) geographical and demographic backgrounds were different from previous studies and (2) the current study enrolled younger participants than the prior studies, such that these individuals were earlier in the process of muscle decline, and exhibited better brain health, motor function, and cognitive function, which were reported to be associated with the presence of HGS asymmetry.

During multiple measurements of HGS, it is possible to maintain the HGS values at relatively stable levels if adequate rest is allowed between tests. Alternatively, the HGS values may be lower initially if the instrument has not been properly adjusted, or they may decrease over time due to fatigue. However, regardless of these circumstances, the average HGS value is generally lower than its maximum value, as supported by the low HGS identification measured using the two calculation methods in this study (refer to S1 and S2 Tables).

The correlation ratio used for detecting HGS asymmetry may vary depending on the changes in the calculation methods between the non-dominant and dominant hands. Our results indicated that there were marked differences between the two methods used for the identification of individuals with HGS asymmetry, with only moderate agreement. A recent study also reported only moderate agreement between the two methods in the identification of weak and asymmetric HGS [20]. Numerous research studies have confirmed that low HGS or asymmetric HGS, either alone or in combination, are associated with poor future health outcomes among older adults [5, 7, 8, 21–24]. Therefore, our findings emphasize the need for a unified approach in defining HGS asymmetry to ensure consistent results across studies and prevent misleading clinical decisions.

The current study is subject to certain limitations. First, all participants were derived from a cohort of Chinese community-dwelling middle-aged and older adults and the results may vary from other populations. Second, the use of HGS values obtained after multiple HGS measurements may offset the differences in HGS asymmetry incidence observed when using the average and maximum methods. However, the original study only conducted two HGS tests, which may explain the differences in results with respect to HGS asymmetry identification. Third, the sample size of individuals over 80 years of age was relatively small, and a larger sample size is needed to confirm our results. Fourth, the physical health status of the participants was primarily evaluated through self-reporting, and included information on chronic diseases, smoking history, drinking history, and other factors. However, the data did not include additional details on the covariates, such as the specific type and quantity of alcohol consumed per week, as well as the frequency and quantity of cigarettes smoked. Moreover, the present study distinguished between asymmetrical HGS and non-asymmetrical HGS, without further analysis of differences in the severity of asymmetrical HGS. Finally, the study was cross-sectional and only explored the differences between the two methods for calculating HGS asymmetry without analysis of the clinical significance of these two methods.

In future research, it would be beneficial to conduct prospective cohort studies that focus on outcomes of clinical significance. These studies should include a large number of participants of all ages and use multiple repetitions of HGS tests. Furthermore, accurate clinical diagnoses should be used, and more detailed groupings based on factors such as the severity of HGS asymmetry or the extent and frequency of smoking/drinking should be created. These measures will help to improve the reliability of research results and provide valuable insights for precise clinical intervention.

## Conclusions

The identification of HGS asymmetry is significantly influenced by the specific method used. The average method identified a higher prevalence of asymmetric HGS in middle-aged and older Chinese individuals. However, this method showed only moderate agreement with the maximum method. These variations underscore the significance of considering the method used for HGS measurement (average or maximum value) when summarizing and analyzing published articles, as well as maintaining consistency in HGS measurement during research.

Currently, there is no standardized method used for determining the value of HGS in cases of asymmetric HGS. The present findings provide evidence of a disparity between the two calculation methods. Therefore, the introduction of a unified protocol may prove beneficial for both research and clinical applications.

## Supporting information

S1 Table Differences in grip strength method identified low HGS. (DOCX)

S2 Table Diagnostic accuracy properties of the different HGS value for HGS to determine HGS weakness. (DOCX)

S1 Data (XLSX)

## References

[1] McGrath R, Vincent BM, Jurivich DA, Hackney KJ, Tomkinson GR, Dahl LJ, et al. Handgrip Strength Asymmetry and Weakness Together Are Associated With Functional Disability in Aging Americans. J Gerontol A Biol Sci Med Sci. 2021;76(2):291–6. doi: 10.1093/gerona/glaa100 32319511

[2] Carson RG. Get a grip: individual variations in grip strength are a marker of brain health. Neurobiol Aging. 2018;71:189–222. doi: 10.1016/j.neurobiolaging.2018.07.023 30172220

[3] McGrath R, Clark BC, Cesari M, Johnson C, Jurivich DA. Handgrip strength asymmetry is associated with future falls in older Americans. Aging Clin Exp Res. 2021;33(9):2461–9. doi: 10.1007/s40520-020-01757-z 33247424 PMC8211412

[4] Baek J, Kim Y, Kim HY. Associations of Handgrip Asymmetry With Impaired Health-Related Quality of Life Among Older Adults in South Korea: A Cross-Sectional Study Using National Survey Data. Asia Pac J Public Health. 2022;34(6–7):649–59. doi: 10.1177/10105395221106629 35730491

[5] Chen Z, Ho M, Chau PH. Handgrip strength asymmetry is associated with the risk of neurodegenerative disorders among Chinese older adults. J Cachexia Sarcopenia Muscle. 2022;13(2):1013–23. doi: 10.1002/jcsm.12933 35178892 PMC8977973

[6] Chen KK, Lee SY, Pang BWJ, Lau LK, Jabbar KA, Seah WT, et al. Associations of low handgrip strength and hand laterality with cognitive function and functional mobility—the Yishun Study. BMC Geriatr. 2022;22(1):677. doi: 10.1186/s12877-022-03363-2 35974301 PMC9382769

[7] Lin S, Wang F, Huang Y, Yuan Y, Huang F, Zhu P. Handgrip strength weakness and asymmetry together are associated with cardiovascular outcomes in older outpatients: A prospective cohort study. Geriatr Gerontol Int. 2022;22(9):759–65. doi: 10.1111/ggi.14451 36058626 PMC9544274

[8] Mahoney SJ, Hackney KJ, Jurivich DA, Dahl LJ, Johnson C, McGrath R. Handgrip Strength Asymmetry Is Associated With Limitations in Individual Basic Self-Care Tasks. J Appl Gerontol. 2022;41(2):450–4. doi: 10.1177/0733464820982409 33356740

[9] Liu M, Liu S, Sun S, Tian H, Li S, Wu Y. Sex Differences in the Associations of Handgrip Strength and Asymmetry With Multimorbidity: Evidence From the English Longitudinal Study of Ageing. J Am Med Dir Assoc. 2022;23(3):493–8.e1. doi: 10.1016/j.jamda.2021.07.011 34389337

[10] McGrath R, Tomkinson GR, LaRoche DP, Vincent BM, Bond CW, Hackney KJ. Handgrip Strength Asymmetry and Weakness May Accelerate Time to Mortality in Aging Americans. J Am Med Dir Assoc. 2020;21(12):2003–7.e1. doi: 10.1016/j.jamda.2020.04.030 32611522

[11] McGrath R, Cawthon PM, Cesari M, Al Snih S, Clark BC. Handgrip Strength Asymmetry and Weakness Are Associated with Lower Cognitive Function: A Panel Study. J Am Geriatr Soc. 2020;68(9):2051–8. doi: 10.1111/jgs.16556 32473060

[12] Chen LK, Woo J, Assantachai P, Auyeung TW, Chou MY, Iijima K, et al. Asian Working Group for Sarcopenia: 2019 Consensus Update on Sarcopenia Diagnosis and Treatment. J Am Med Dir Assoc. 2020;21(3):300–7.e2. doi: 10.1016/j.jamda.2019.12.012 32033882

[13] MacDermid J, Solomon G, Valdes K. Clinical assessment recommendations: American Society of Hand Therapists; 2015.

[14] Choi JY, Lee S, Min JY, Min KB. Asymmetrical Handgrip Strength Is Associated with Lower Cognitive Performance in the Elderly. J Clin Med. 2022;11(10). doi: 10.3390/jcm11102904 35629029 PMC9144314

[15] Parker K, Rhee Y, Tomkinson GR, Vincent BM, O’Connor ML, McGrath R. Handgrip Weakness and Asymmetry Independently Predict the Development of New Activity Limitations: Results from Analyses of Longitudinal Data from the US Health and Retirement Study. J Am Med Dir Assoc. 2021;22(4):821–6.e1. doi: 10.1016/j.jamda.2020.11.006 33290729

[16] Gómez-Campos R, Vidal Espinoza R, de Arruda M, Ronque ERV, Urra-Albornoz C, Minango JC, et al. Relationship between age and handgrip strength: Proposal of reference values from infancy to senescence. Front Public Health. 2022;10:1072684. doi: 10.3389/fpubh.2022.1072684 36777772 PMC9909206

[17] McGrath R, Cawthon PM, Clark BC, Fielding RA, Lang JJ, Tomkinson GR. Recommendations for Reducing Heterogeneity in Handgrip Strength Protocols. J Frailty Aging. 2022;11(2):143–50. doi: 10.14283/jfa.2022.21 35441190

[18] Verghese J, Wang C, Bennett DA, Lipton RB, Katz MJ, Ayers E. Motoric cognitive risk syndrome and predictors of transition to dementia: A multicenter study. Alzheimers Dement. 2019;15(7):870–7. doi: 10.1016/j.jalz.2019.03.011 31164315 PMC6646063

[19] McGrath R, Lang JJ, Ortega FB, Chaput JP, Zhang K, Smith J, et al. Handgrip strength asymmetry is associated with slow gait speed and poorer standing balance in older Americans. Arch Gerontol Geriatr. 2022;102:104716. doi: 10.1016/j.archger.2022.104716 35569287

[20] Weng M, Pu J, Wang B, Wang Y. Risk factors associated with weak and asymmetric handgrip strength in older Chinese adults. Am J Hum Biol. 2023:e24007. doi: 10.1002/ajhb.24007 37867368

[21] Dowling L, Cuthbertson DJ, Walsh JS. Reduced muscle strength (dynapenia) in women with obesity confers a greater risk of falls and fractures in the UK Biobank. Obesity (Silver Spring). 2023;31(2):496–505. doi: 10.1002/oby.23609 36504327 PMC10108064

[22] Go YJ, Lee DC, Lee HJ. Association between handgrip strength asymmetry and falls in elderly Koreans: A nationwide population-based cross-sectional study. Arch Gerontol Geriatr. 2021;96:104470. doi: 10.1016/j.archger.2021.104470 34243024

[23] Li R, Xia J, Zhang XI, Gathirua-Mwangi WG, Guo J, Li Y, et al. Associations of Muscle Mass and Strength with All-Cause Mortality among US Older Adults. Med Sci Sports Exerc. 2018;50(3):458–67. doi: 10.1249/MSS.0000000000001448 28991040 PMC5820209

[24] Klawitter L, Vincent BM, Choi BJ, Smith J, Hammer KD, Jurivich DA, et al. Handgrip Strength Asymmetry and Weakness Are Associated With Future Morbidity Accumulation in Americans. J Strength Cond Res. 2022;36(1):106–12. doi: 10.1519/JSC.0000000000004166 34941610

