# Supplementary material for: The accuracy of different calculation methods when identifying handgrip strength asymmetry among middle-aged and older Chinese adults
Source: PLoS One. 2024 Mar 28;19(3):e0299469. doi: 10.1371/journal.pone.0299469 (PMC10977670; doi:10.1371/journal.pone.0299469)
Supplement: S1 Table — (DOCX) [file pone.0299469.s001.docx]

**S1 Table** **Differences in grip strength method identified low HGS.**

| **Variables** | **Maximum value of both hands** | | **P-value** |
| --- | --- | --- | --- |
|  | **Normal HGS** | **Low HGS** |  |
| **Male*** |  |  |  |
| **Average value of both hands** |  |  | ＜0.001 |
| Normal HGS | 1690（100) | 0 |  |
| Low HGS | 341(25.3) | 1006(74.7) |  |
| **Maximum value of the dominant hand** |  |  | ＜0.001 |
| Normal HGS | 1943(100) | 0 |  |
| Low HGS | 88(8) | 1006(92） |  |
| **Average value of** **the dominant hand** |  |  | ＜0.001 |
| Normal HGS | 1784（100) | 0 |  |
| Low HGS | 247(19.7) | 1006(80.3) |  |
| **Male**** |  |  |  |
| **Average value of both hands** |  |  | ＜0.001 |
| Normal HGS | 1347(100) | 0 |  |
| Low HGS | 329(26.7) | 903(73.3) |  |
| **Maximum value of the dominant hand** |  |  | ＜0.001 |
| Normal HGS | 1605(100) | 0 |  |
| Low HGS | 71(7.3) | 903(92.7) |  |
| **Average value of the dominant hand** |  |  | ＜0.001 |
| Normal HGS | 1457(100) | 0 |  |
| Low HGS | 219(19.5) | 903(80.5) |  |
| **Male***** |  |  |  |
| **Average value of both hands** |  |  | ＜0.001 |
| Normal HGS | 96(100) | 0 |  |
| Low HGS | 20(25.3) | 59(74.7) |  |
| **Maximum value of the dominant hand** |  |  | ＜0.001 |
| Normal HGS | 111(100) | 0 |  |
| Low HGS | 5(7.8) | 59(92.2) |  |
| **Average value of the dominant hand** |  |  | ＜0.001 |
| Normal HGS | 103(100) | 0 |  |
| Low HGS | 13(18.1) | 59(81.9) |  |
| **Female*** |  |  |  |
| **Average value of both hands** |  |  | ＜0.001 |
| Normal HGS | 3253(100) | 0 |  |
| Low HGS | 180(46) | 211(54) |  |
| **Maximum value of the dominant hand** |  |  | ＜0.001 |
| Normal HGS | 3377(100) | 0 |  |
| Low HGS | 56(21) | 211(79) |  |
| **Average value of the dominant hand** |  |  | ＜0.001 |
| Normal HGS | 3312(100) | 0 |  |
| Low HGS | 121(36.4) | 211(63.8) |  |
| **Female**** |  |  |  |
| **Average value of both hands** |  |  | ＜0.001 |
| Normal HGS | 2232(100) | 0 |  |
| Low HGS | 121(43.4) | 158(56.6) |  |
| **Maximum value of the dominant hand** |  |  | ＜0.001 |
| Normal HGS | 2320(100) | 0 |  |
| Low HGS | 33(17.3) | 158(82.7) |  |
| **Average value of the dominant hand** |  |  | ＜0.001 |
| Normal HGS | 2265(100) | 0 |  |
| Low HGS | 88(35.8) | 158(64.2) |  |
| **Female***** |  |  |  |
| **Average value of both hands** |  |  | ＜0.001 |
| Normal HGS | 200(100) | 0 |  |
| Low HGS | 11(64.7) | 6(35.3) |  |
| **Maximum value of the dominant hand** |  |  | ＜0.001 |
| Normal HGS | 211(100) | 0 |  |
| Low HGS | 0 | 6(100) |  |
| **Average value of the dominant hand** |  |  | ＜0.001 |
| Normal HGS | 204(100) | 0 |  |
| Low HGS | 7(53.8) | 6(46.2) |  |

Note:*45≤age＜60

**60≤age＜80

***80≤age
