# Supplementary material for: The accuracy of different calculation methods when identifying handgrip strength asymmetry among middle-aged and older Chinese adults
Source: PLoS One. 2024 Mar 28;19(3):e0299469. doi: 10.1371/journal.pone.0299469 (PMC10977670; doi:10.1371/journal.pone.0299469)
Supplement: S2 Table — (DOCX) [file pone.0299469.s002.docx]

**S2 Table** **Diagnostic accuracy properties of the different HGS value for HGS to determine HGS weakness.**

| **Variables** | **Cutoff** | **Specificity** | **Sensitivity** | **AUC** | **PPV** | **NPV** | **Kappa value** |
| --- | --- | --- | --- | --- | --- | --- | --- |
| **Male*** |  |  |  |  |  |  |  |
| Maximum value of both hands | 28 | Ref. | Ref. | Ref. | Ref. | Ref. | Ref. |
| Average value of both hands | 28 | 83.2% | 100% | 0.992（0.99-0.994） | 0.75 | 1 | 0.767 |
| Maximum value of the dominant hand | 28 | 95.7% | 100% | 0.991（0.989-0.994） | 0.92 | 1 | 0.936 |
| Average value of the dominant hand | 28 | 87.8% | 100% | 0.988（0.985-0.991） | 0.8 | 1 | 0.827 |
| **Male**** |  |  |  |  |  |  |  |
| Maximum value of both hands | 28 | Ref. | Ref. | Ref. | Ref. | Ref. | Ref. |
| Average value of both hands | 28 | 80.4% | 100% | 0.991（0.988-0.933） | 0.73 | 1 | 0.741 |
| Maximum value of the dominant hand | 28 | 95.8% | 100% | 0.991（0.988-0.994） | 0.93 | 1 | 0.941 |
| Average value of the dominant hand | 28 | 86.9% | 100% | 0.988（0.984-0.991） | 0.8 | 1 | 0.823 |
| **Male***** |  |  |  |  |  |  |  |
| Maximum value of both hands | 28 | Ref. | Ref. | Ref. | Ref. | Ref. | Ref. |
| Average value of both hands | 28 | 82.8% | 100% | 0.996（0.99-1） | 0.75 | 1 | 0.764 |
| Maximum value of the dominant hand | 28 | 95.7% | 100% | 0.995（0.99-1） | 0.92 | 1 | 0.937 |
| Average value of the dominant hand | 28 | 88.8% | 100% | 0.993（0.985-1） | 0.82 | 1 | 0.842 |
| **Female*** |  |  |  |  |  |  |  |
| Maximum value of both hands | 18 | Ref. | Ref. | Ref. | Ref. | Ref. | Ref. |
| Average value of both hands | 18 | 94.8% | 100% | 0.997(0.996-0.998) | 0.54 | 1 | 0.677 |
| Maximum value of the dominant hand | 18 | 98.4% | 100% | 0.996(0.994-0.997) | 0.79 | 1 | 0.875 |
| Average value of the dominant hand | 18 | 96.5% | 100% | 0.995(0.993-0.997) | 0.64 | 1 | 0.76 |
| **Female**** |  |  |  |  |  |  |  |
| Maximum value of both hands | 18 | Ref. | Ref. | Ref. | Ref. | Ref. | Ref. |
| Average value of both hands | 18 | 94.9% | 100% | 0.998(0.997-0.999) | 0.57 | 1 | 0.699 |
| Maximum value of the dominant hand | 18 | 98.6% | 100% | 0.997(0.995-0.998) | 0.83 | 1 | 0.898 |
| Average value of the dominant hand | 18 | 96.2% | 100% | 0.996(0.994-0.997) | 0.64 | 1 | 0.764 |
| **Female***** |  |  |  |  |  |  |  |
| Maximum value of both hands | 18 | Ref. | Ref. | Ref. | Ref. | Ref. | Ref. |
| Average value of both hands | 18 | 94.8% | 100% | 0.996（0.989-1） | 0.35 | 1 | 0.501 |
| Maximum value of the dominant hand | 18 | 100% | 100% | 1(1-1) | 1 | 1 | 1 |
| Average value of the dominant hand | 18 | 96.7% | 100% | 0.998(0.994-1) | 0.46 | 1 | 0.617 |

Note:*45≤age＜60

**60≤age＜80

***80≤age
